# Real-Time Imaging of Platelet-Initiated Plasma Clot Formation and Lysis Unveils Distinct Impacts of Anticoagulants

**DOI:** 10.1055/a-2497-4213

**Published:** 2025-01-09

**Authors:** Yuko Suzuki, Nitty S. Mathews, Hideto Sano, Nanami Morooka, Naoki Honkura, Tetsumei Urano

**Affiliations:** 1 Department of Medical Physiology, Hamamatsu University School of Medicine, Hamamatsu, Japan; 2 Department of Transfusion Medicine & Immunohematology, Christian Medical College Vellore, India; 3 Department of Physiology, Tokai University School of Medicine, Kanagawa, Japan; 4 Shizuoka Graduate University of Public Health, Shizuoka, Japan

**Keywords:** anticoagulants, confocal microscopy, fibrinolysis, platelets, thrombin-activatable fibrinolysis inhibitor

## Abstract

**Background:**

Fibrinolysis is spatiotemporally well-regulated and greatly influenced by activated platelets and coagulation activity. Our previous real-time imaging analyses revealed that clotting commences on activated platelet surfaces, resulting in uneven-density fibrin structures, and that fibrinolysis initiates in dense fibrin regions and extends to the periphery. Despite the widespread clinical use of direct oral anticoagulants (DOACs), their impact on thrombin-dependent activation of thrombin-activatable fibrinolysis inhibitor (TAFI) and fibrinolysis remains unclear. Here, we investigated the effects of different DOACs on the TAFI-mediated inhibition of fibrinolysis.

**Methods:**

Using human platelet-containing plasma, we performed turbidimetric assays, thrombin generation assays, and confocal laser scanning microscopy to assess the effects of anticoagulants on fibrinolysis.

**Results and Conclusion:**

Activated platelets-prolonged plasma clot lysis time, shortened by activated TAFI inhibitor (TAFIaI), positively correlated with the amount of thrombin generated. Rivaroxaban (an activated factor X inhibitor) and dabigatran (a direct thrombin inhibitor) dose-dependently shortened lysis time comparably. The highest concentration of DOACs showed no further shortening of lysis time with TAFIaI. The fibrin network structures initiated by activated platelets and the localization of fluorescently labeled plasminogen were unique for these two drugs. Rivaroxaban maintained an uneven fibrin network but promoted faster plasminogen accumulation and fibrinolysis from outside dense fibrin regions. Conversely, dabigatran resulted in a more even fibrin network, with fibrinolysis starting from the activated platelets and propagating to the periphery. Visualizing and analyzing the patterns of fibrin network formation, plasminogen accumulation, and fibrinolysis provide new insights into the specific impact of anticoagulants on coagulation and fibrinolysis.

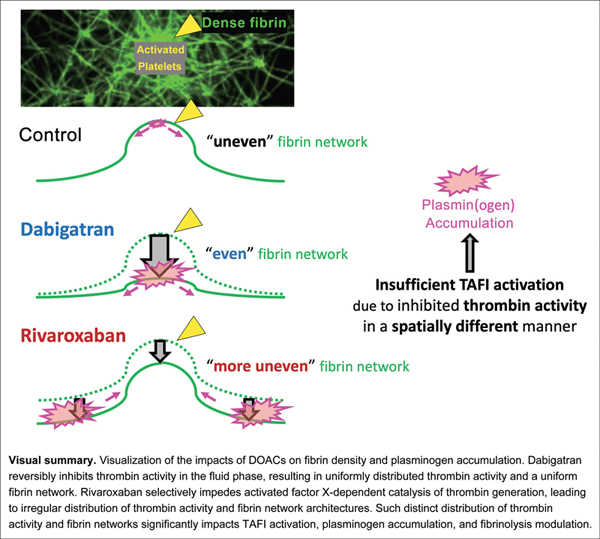

## Introduction

Direct oral anticoagulants (DOACs) are widely used to prevent and treat thromboembolic diseases due to their specificity in targeting coagulation factors. 1 2 They offer several advantages over vitamin K antagonists, such as not requiring regular coagulation monitoring and fewer drug interactions. Dabigatran, a direct thrombin inhibitor (DTI), and the direct activated factor X (FXa) inhibitors (FXaIs), such as rivaroxaban, apixaban, and edoxaban, are available for various clinical indications. Anticoagulants still pose a bleeding risk, but DOACs, owing to their lower risk of major bleeding, are generally preferred over vitamin K antagonists, with some exceptions. 1 3 Clinical and observational studies have compared the efficacy and safety profiles of different classes of anticoagulants in patients with atrial fibrillation. 4 5 6 7 8 These studies have revealed variations in bleeding risk among different medications, which may be varied not only by the overall anticoagulatory properties of the medications but also by their subsequent impacts on fibrinolysis.

Fibrinolysis plays a crucial role in dissolving blood clots containing fibrin by activating plasminogen into plasmin. Appropriate fibrinolysis relies on the dynamic reactions between activators and inhibitors regulating both plasminogen activation and plasmin activity. 9 Tissue-type plasminogen activator (tPA) initiates plasminogen activation by forming a trimeric complex with plasminogen on fibrin. Plasminogen interacts with fibrin primarily through its carboxy-terminal lysine residues (C-ter Lys), which are susceptible to cleavage by activated thrombin-activatable fibrinolysis inhibitor (TAFIa, known as carboxypeptidase B2), a natural fibrinolysis inhibitor. 10 11 Although plasmin and thrombin are known to activate TAFI, 10 12 robust activation of plasma TAFI takes upon the binding of thrombin to thrombomodulin, which is substantially expressed on vascular endothelial cells. In addition to the plasma membrane-penetrating forms, 13 soluble forms of thrombomodulin 14 15 have the potential to enhance thrombin-dependent activation of TAFI. Thus, the role of TAFIa in inhibiting fibrinolysis becomes significant when thrombin activity reaches sufficient levels.

Thus, thrombin activity plays a crucial role in the regulation of fibrinolysis. Diminished thrombin activity resulting from coagulation factor deficiencies 16 17 18 19 or antithrombotic treatment 20 21 22 23 24 25 significantly affects TAFI activation. Inadequate levels of TAFIa are considered to limit hemostatic thrombus stability, exacerbating bleeding symptoms. Unlike FXaIs, DTIs demonstrate profibrinolytic activity in plasma samples from patients undergoing anticoagulant treatment. 22 25 This difference is attributable to their distinct effects on fibrin formation, including the thrombin generation curve. Although the profibrinolytic effect of FXa inhibition has been reported, 20 24 26 no significant variations in clot lysis time were observed in samples from anticoagulant-treated patients compared with control samples. 23 Therefore, a comprehensive understanding of the delicate balance between coagulation and fibrinolytic properties is crucial.

Real-time visualization of platelet-containing plasma using laser-scanning confocal microscopy provides valuable insights into the spatial and temporal mechanisms regulating thrombus formation and subsequent lysis. Compelling evidence suggests that activated platelets, by exposing phosphatidylserine (PS) on their outer membrane surface, play a pivotal role in initiating and enhancing both the formation and lysis of fibrin networks. 27 28 Our recent study further elucidated the significance of soluble thrombomodulin in plasma, revealing its role in activating TAFI and attenuating fibrinolysis in an in vitro platelet-containing plasma clot lysis assay. 14 Moreover, using a mouse microthrombus model via intravital two-photon microscopy, we revealed that activation of TAFI results in a reduction in fibrinolysis, 29 potentially mediated by endogenously expressed thrombomodulin.

In this study, we explored the influence of activated platelet-enhanced thrombin generation on TAFI activation, unraveling distinct effects of DTI and FXaI on both coagulation and fibrinolysis. By employing real-time imaging analysis in spatial–temporal dimensions with platelet-containing plasma, we conducted a comprehensive evaluation of fibrin network formation, the propagation of plasminogen accumulation, and subsequent lysis expansion. This innovative approach provides valuable insights into the differential effects of DTIs and FXaIs, elucidating their respective influences on fibrinolysis.

## Materials and Methods

### Reagents

Rivaroxaban and dabigatran (BIBR 953) were purchased from Chem Scene, LLC (Monmouth Junction, NJ, United States) and Selleck Biotech (Tokyo, Japan), respectively. The TAFIa inhibitor (DS45251085, a DS-1040 analog, (2Z)-5-amino-2-({1-[(1r,4r)-4-methylcyclohexyl]-1H-imidazol-4-yl}methyl)pent-2-enoic acid; WO2013039202) was kindly provided by Daiichi Sankyo Co., Ltd. (Tokyo, Japan). Recombinant tissue-type plasminogen activator (rt-PA), Dade Innovin human recombinant tissue factor, monoclonal antibody against human thrombomodulin (CD141; thrombomodulin-neutralizing antibody [aTM-Ab]), human fibrinogen, and type B gelatin were purchased from Mitsubishi Tanabe Pharma Corporation (Osaka, Japan), Siemens Healthcare Diagnostics (Deerfield, IL, United States), Enzyme Research Laboratories (South Bend, IN, United States), Hycult Biotech (Uden, The Netherlands), and Sigma-Aldrich Inc (St. Louis, MO, United States), respectively. Alexa Fluor (AF) fluorescent dyes were obtained from Life Technologies Corporation (Eugene, OR, United States). Human Glu-plasminogen was purified from freshly frozen human plasma (Japanese Red Cross Society, Tokyo, Japan) using affinity chromatography on sepharose-lysine.

### Plasma Preparation

Blood samples were collected from healthy individuals using tubes containing 0.1 volumes of 3.2% trisodium citrate. The samples were then centrifuged at 250 × g for 10 minutes at 22°C to obtain platelet-rich plasma (PRP). The platelet concentration was determined using a whole-blood cell counter, pocH-80i (Sysmex Corporation, Kobe, Japan). Platelet-free plasma (PFP) was prepared by centrifuging the samples at 1,800 × g for 10 minutes at 4°C, followed by subsequent centrifugation at 3,000 × g for 10 minutes at 4°C. We confirmed that PFP was platelet-free by counting them using pocH-80i. For the experiments, we diluted PRP with PFP to adjust the platelet counts to 4.0/8.0 × 10 ^4^ platelets/µL. All the samples were processed within 6 hours of blood collection. The study was conducted in accordance with the principles of the Declaration of Helsinki and was approved by the Ethics Committee of Hamamatsu University School of Medicine (No. 16–286). All participants provided written informed consent.

### Turbidity Assay

The turbidimetric assay was performed using a 96-well microtiter plate. Plasma samples, with or without platelets, were diluted to a final concentration of 50% using 10 mM HEPES buffer (pH 7.4, HBS; 140 mM NaCl, 5 mM KCl, and 1 mM MgCl _2_ ). tPA (1.5 nM) was added to initiate fibrinolysis. If needed, 5 µM TAFIa inhibitor, 10 µg/mL aTM-Ab, and different concentrations of dabigatran/rivaroxaban were mixed. Coagulation was initiated by adding 10 mM CaCl _2_ and Dade Innovin (diluted to 1:3,000). The plate was maintained at 37°C, and the turbidity was measured every minute at a wavelength of 405 nm using a Multiskan FC spectrophotometer (Thermo Fisher Scientific, Waltham, MA, USA). Clotting time was determined as the time taken for the turbidity to increase halfway during clot formation. Lysis time was defined as the time between the halfway increase in turbidity during clot formation and the halfway decrease during clot lysis.

### Thrombin Generation Assay

Thrombin generation assay developed by Hemker et al 30 was performed following the instruction procedure outlined in the Technothrombin® TGA kit (Technoclone GmbH, Vienna, Austria), with slight modifications to previously established methods. 31 We decided not to include phospholipids in the assay, as they are naturally supplied by the activated platelets, along with other physiologically active substances contained within the platelets in our experimental configuration. This approach may allow a more natural reproduction of the coagulation cascade. The thrombin calibration curve was recorded using the calibrator (Technothrombin® TGA CAL) with a fluorogenic substrate of thrombin (Z-G-G-R-AMC; Technothrombin® TGA SUB) at a concentration of 100 µM. Thrombin generation in plasma samples was determined under similar conditions to the turbidimetric assay, except for the inclusion of tPA. Briefly, 50 µL (100 µL in final volume) of plasma containing 0, 4.0, or 8.0 × 10 ^4^ platelets/µL and Dade Innovin diluted to 1:3,000 were added to the well of a black 96-well plate (Thermo Fisher Scientific, Roskilde, Denmark). The samples were then incubated, if necessary, with 5 µM TAFIa inhibitor, 10 µg/mL aTM-Ab, and different concentrations of dabigatran/rivaroxaban for 10 minutes at 37°C. A mixture of CaCl _2_ (10 mM) and a fluorogenic substrate (100 µM) was added. The development of fluorescence intensity was measured every minute for 60 minutes using a BioTek Synergy H1 microplate reader (Agilent Technologies, Santa Clara, CA, United States) with excitation and emission filter sets of 360 and 460 nm, respectively. The first derivative of the reaction curve was calculated, and thrombin generation was determined using a calibration curve. Under specific circumstances, such as in platelet-free conditions, thrombin generation was limited due to the insufficient availability of phospholipids. Therefore, the endogenous thrombin potential at 60 minutes (ETP-60) was evaluated by calculating the area under the curve of thrombin generation at 60 minutes.

### Confocal Imaging

We used a confocal laser scanning microscope, TCS SP8 (Leica Microsystems GmbH, Wetzlar, Germany), equipped with a 20× (NA 0.8) objective lens and a stage top incubator (Tokai Hit Co., Ltd., Shizuoka, Japan) to maintain the samples at a temperature of 37°C. All experiments were conducted in a 0.1% gelatin-coated, 35-mm glass base dish (AGC Inc., Tokyo, Japan). We supplemented a total of 4.0 × 10 ^4^ platelets/µL in half-diluted plasma with tPA (2 nM), trace amounts of AF 488-labeled fibrinogen (fbg-488), and AF 568-labeled plasminogen (plg-568). The samples were then incubated for 5 minutes on a heating stage at 37°C. Then, a solution containing 10 mM CaCl _2_ and Dade Innovin, diluted to 1:3,000, was added. Image capturing was initiated immediately. The capture process involved scanning three focal planes at 1-μm intervals over a duration of 1 minute. These three planes were then stacked to create a single image.

### Fluorescence Intensity Analysis

Fluorescence intensity analysis was conducted using ImageJ (v2.0.0-rc-69/1.52n). The fluorescence intensities of fbg-488 and plg-568 at the five concentric circles from the center of the dense fibrin region ( Supplementary Fig. S1A , available in the online version) were measured separately. We defined the fibrin fiber appearance time ( Supplementary Fig. S1A , [available in the online version], indicated by the green arrow in the graph) as the marker of coagulation activity based on the changes in fbg-488 fluorescence intensity in the central region. The plasminogen accumulation time was determined by measuring the time from the appearance of fibrin fibers to the point of maximum intensity of plg-568 fluorescence in the central region ( Supplementary Fig. S1A [available in the online version], between the green and red arrows shown by the duration of the double-headed arrow in the graph). This time served as an indicator of TAFI activity. 14 Supplementary Fig. S1B–C (available in the online version) shows representative changes in fbg-488 and plg-568 fluorescence intensities in five concentric circles. To analyze the density of fibrin and the propagation of fibrin formation, the relative green fluorescence intensity of the outer regions (C1 to C4 indicating a more peripheral region) was calculated by comparing it with the maximum intensity at the center (C0). The propagation of plasminogen accumulation was assessed by measuring the delay in reaching the maximum values at five different regions, moving from the center to the periphery, and labeled as delay 1 (d1) to delay 4 (d4).

### Statistics

All data were analyzed using Statcel 3 (v3), an add-in software for Microsoft Excel (for Mac 2011 v14.7.7) (OMS Publishing Inc., Saitama, Japan), operated by MacBook Pro running macOS Mojave v10.14.3. The specific methods used are described in the figure legends.

## Results

### Activated Platelets Enhance Thrombin Generation, Followed by TAFI Activation and Fibrinolysis Inhibition

**Fig. 1:**
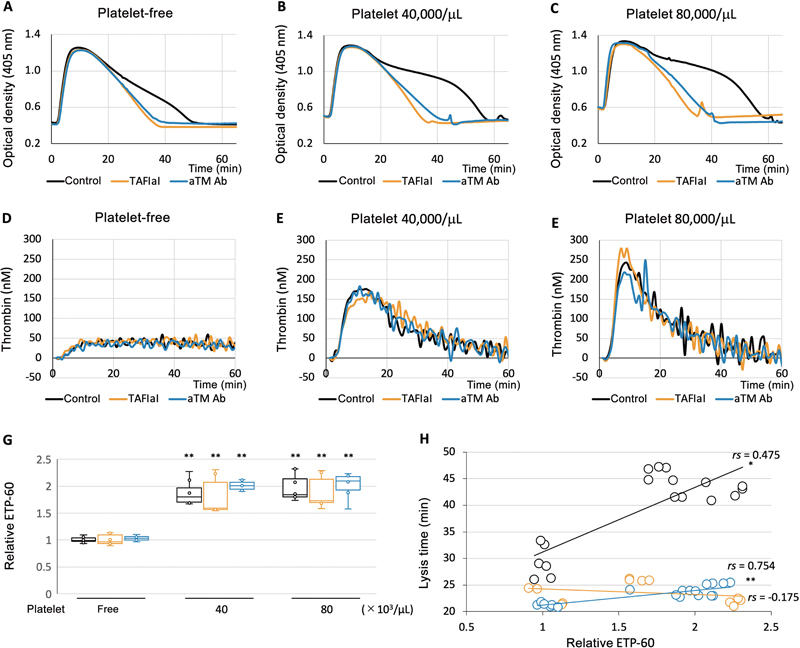
Activated platelets potentiate thrombin generation and thrombin-activatable fibrinolysis inhibitor (TAFI) activation. The representative data show changes in turbidity during the formation and lysis of clots in plasma with and without platelets ( **A–C** ) and during thrombin generation ( **D–F** ). Assays were conducted with an activated TAFI inhibitor (TAFIaI: orange lines) or anti-thrombomodulin-neutralizing antibody (aTM Ab; blue lines), in addition to the control without any inhibitor or antibody (black lines). ( **G** ) The relative endogenous thrombin potential (ETP)-60 of the platelet-free control was calculated for each group (control: black boxes; TAFIaI: orange boxes; aTM Ab: blue boxes), using six to eight samples from three individuals. The data are shown as median and interquartile ranges and were analyzed using a nonparametric multiple comparison test, specifically the Shirley–Williams method (** *p* < 0.01). ( **H** ) The relationship between ETP-60 and lysis time (control: black circles; TAFIaI: orange circles; aTM Ab: blue circles) was analyzed using Spearman's correlation coefficient by rank test ( *rs* : * *p* < 0.05, ** *p* < 0.01). The different groups are represented by the following colors: black: control ( *N* = 18); orange: TAFIaI ( *N* = 12); and blue: aTM Ab ( *N* = 19).

In our recent study, we revealed that activated platelets significantly prolonged clot lysis time. 14 This phenomenon is shown as a control and was effectively mitigated by either a TAFIa inhibitor or an aTM-Ab ( Fig. 1A–C ). Under comparable conditions, thrombin activity was manually monitored using a fluorogenic substrate specific to thrombin in the absence of tPA. Fig. 1D–F displays representative curves of thrombin concentrations calculated at varying platelet counts, highlighting the augmented thrombin generation facilitated by platelets. Due to the challenges in determining typical parameters of the modified thrombin generation assay used in this study, only the ETP-60 was analyzed as relative values to the control under platelet-free conditions. The presence of platelets led to a notable increase in the relative ETP-60 levels compared to its absence ( Fig. 1G ). Inhibition of TAFI activity or its activation, either through a TAFIa inhibitor or an aTM-Ab, did not affect relative ETP-60. In the control group, a positive correlation was noted between lysis time and relative ETP-60; however, this correlation disappeared when the TAFIa inhibitor was added ( Fig. 1H ). In the presence of aTM-Ab, a stronger correlation was observed than that in the control group, despite a much shorter clot lysis time.

### DTI and FXaI Have Different Effects on Thrombin Generation and Clotting Time

**Fig. 2:**
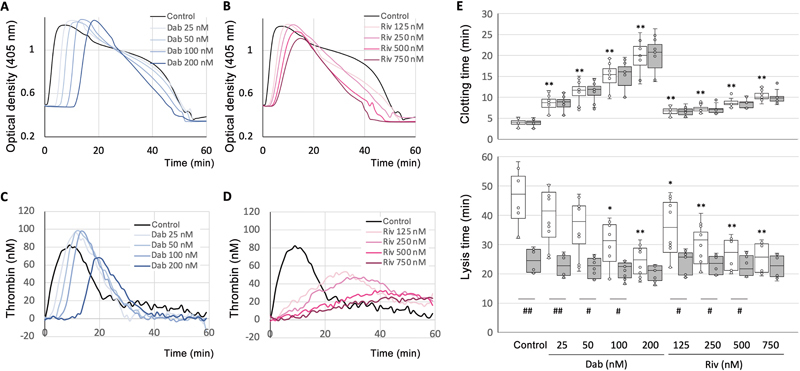
Anticoagulant-induced reduction of thrombin activity leads to shorter lysis time due to inadequate thrombin-activatable fibrinolysis inhibitor (TAFI) activation. The representative data show changes in turbidity ( **A, B** ) and thrombin generation ( **C, D** ) in platelet-containing plasma treated with different concentrations of dabigatran (Dab 25, 50, 100, and 200 nM; represented by blue lines in **A** , **C** ) or rivaroxaban (Riv 125, 250, 500, and 750 nM; represented by pink lines in **B** , **D** ). The black lines indicate the absence of anticoagulants (control). The thicker pink and blue lines indicate higher anticoagulant concentrations. In panel **E** , the clotting time (upper panel) and lysis time (lower panel) are shown for the control and different concentrations of anticoagulants in platelet-containing plasma. These data include 15 samples from three individuals. The data are presented as the median and interquartile ranges, with or without activated TAFI (TAFIa) inhibitor, indicated by closed and open bars, respectively. Statistical analysis was performed using the Mann–Whitney U-test (# *p* < 0.05, ## *p* < 0.01). The dose-dependent effect of anticoagulants in the absence of TAFIa inhibitor was analyzed using the Shirley–Williams method, a nonparametric multiple comparison test (* *p* < 0.05, ** *p* < 0.01).

The positive correlation between thrombin generation and clot lysis time led us to investigate the effect of reduced thrombin activity on clot lysis time. We examined the effects of two types of anticoagulants, dabigatran (a DTI) and rivaroxaban (an FXaI), in the *in-vitro* experiment. The concentrations used here refer to plasma concentrations after administration to healthy volunteers 32 and patients with atrial fibrillation. 22 Dabigatran (25–200 nM) and rivaroxaban (125–750 nM) exhibited concentration-dependent patterns in the turbidity curves of plasma clot formation and lysis ( Fig. 2A, B ), as well as in thrombin generation ( Fig. 2C, D ). As reviewed earlier, 33 dabigatran delayed the onset of thrombin generation but had minimal effect on peak values ( Fig. 2C ). Conversely, rivaroxaban tended to flatten the curve and prolong the reaction ( Fig. 2D ). These differences in the thrombin generation led to variations in clotting times, measured as the time from half-increasing turbidity. This was evident in the case of rivaroxaban, which demonstrated a shorter clotting time than dabigatran ( Fig. 2E , upper, open bars).

### Reduction of Thrombin Activity Directly Affects TAFI Activation

Compared with dabigatran, clotting time was less prolonged with rivaroxaban, but the concentration-dependent shortening of lysis time with rivaroxaban was more significant than that with dabigatran ( Fig. 2E , lower, open bars). The lysis time exhibited significant differences in the absence ( Fig. 2E , open bars) or presence ( Fig. 2E , closed bars) of the TAFIa inhibitor, but these differences disappeared at the highest concentrations of both anticoagulants. This suggests that the reduction of thrombin generation/activity by higher concentrations of anticoagulants leads to insufficient TAFI activation and subsequent fibrinolysis inhibition. The TAFIa inhibitor had no effect on clotting time.

**Fig. 3:**
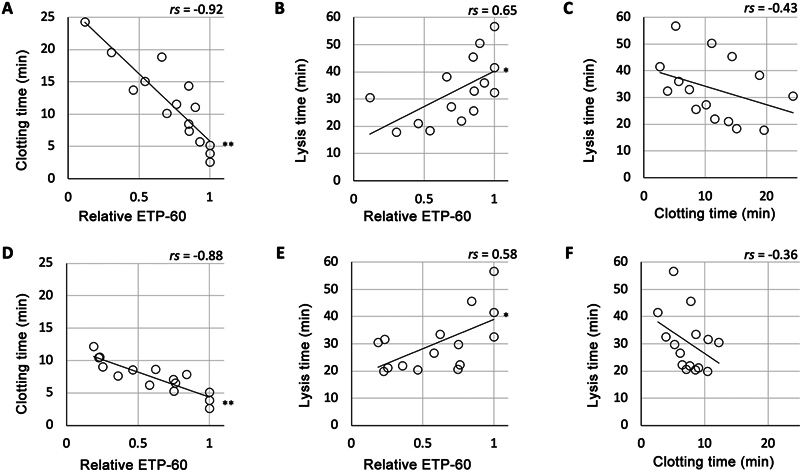
Correlation between coagulation and fibrinolysis parameters in platelet-containing plasma treated with different classes of anticoagulants. The correlations among relative endogenous thrombin potential (ETP)-60, clotting time, and lysis time in platelet-containing plasma treated with dabigatran ( **A–C** ) or rivaroxaban ( **D–F** ) were analyzed using Spearman's correlation coefficient (shown as *rs* in graphs) by a rank test (15 samples from three individuals, * *p* < 0.05, ** *p* < 0.01).

To determine the exact effect of coagulation activity on TAFI activation, we conducted an analysis to elucidate the interplay among clotting time, thrombin generation (represented by relative ETP-60), and lysis time. We observed a strong negative correlation between clotting time and relative ETP-60, as well as a moderate positive correlation between lysis time and relative ETP-60, for both anticoagulants ( Fig. 3A–C : dabigatran; Fig. 3D–F : rivaroxaban). However, we found no significant correlation between clotting and lysis times.

### Real-Time Imaging Analysis of Clot Formation and Lysis in Platelet-Containing Plasma

**Fig. 4:**
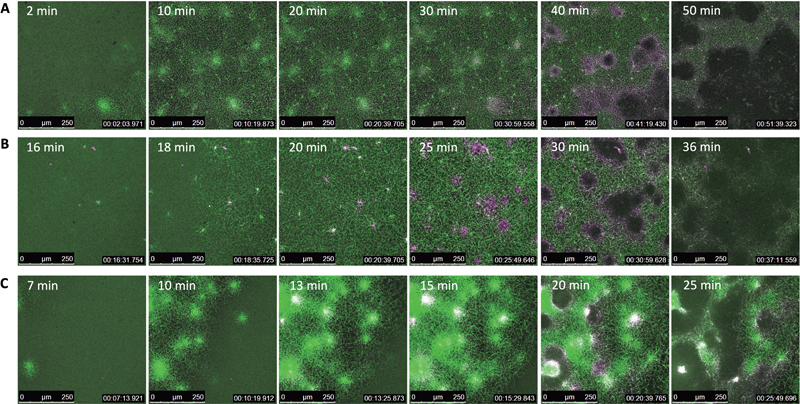
Real-time imaging analysis of clot formation and dissolution in platelet-containing plasma. We used a confocal laser scanning microscope (TCS SP8; Leica Microsystem GmbH), equipped with a 20× (NA 0.8) objective lens and a stage top incubator (Tokai Hit Co., Ltd.), maintaining samples at a temperature of 37°C. Coagulation and fibrinolysis of platelet-containing plasma were initiated by tissue factor and tissue-type plasminogen activator, respectively. Trace amounts of Alexa Fluor (AF) 488-labeled fibrinogen (green) and AF 568-labeled plasminogen (magenta) were added for visualization. Representative sequentially overlaid images are shown. The dense fibrin network at the activated platelets indicates the heterogeneity of the fibrin network structure. ( **A** ) Control (2, 10, 20, 30, 40, and 50 minutes after the start of video capture). ( **B** ) 200 nM dabigatran (16, 18, 20, 25, 30, and 36 minutes after the start of video capture). ( **C** ) 500 nM rivaroxaban (7, 10, 13, 15, 20, and 25 minutes after the start of video capture).

We examined the spatial distribution of fbg-488 and plg-568 during the formation and subsequent lysis of clots oriented by activated platelets. Fig. 4 illustrates the process from the initial formation of the fibrin network to its lysis of more than 50%. These images reveal the significant differences between DOACs in the distribution of the fibrin network, accumulation of plasminogen, and the subsequent lysis process. In the absence of anticoagulants (control; Fig. 4A ), we observed the formation and propagation of dense fibrin regions through activated platelets, as demonstrated in the previous study. 28 Treatment with 200 nM dabigatran, even at a low concentration of 100 nM ( Supplementary Fig. S2 , available in the online version), reduced the formation of dense fibrin regions ( Fig. 4B ). In contrast, treatment with 500 nM rivaroxaban resulted in larger and more intense dense fibrin regions ( Fig. 4C ).

In control, increased accumulation of plasminogen in dense fibrin regions was observed, followed by fibrinolysis, indicated by enhanced red fluorescence of plg-568 ( Fig. 4A ). The patterns of plasminogen accumulation and lysis remained consistent between the control and 200 nM dabigatran ( Fig. 4A, B ), but these were not sufficient to extend from activated platelets to the periphery at low dabigatran concentrations ( Supplementary Fig. S2 , available in the online version). Notably, distinct variations in plasminogen accumulation and lysis dynamics were observed with rivaroxaban ( Fig. 4C ), in contrast to patterns observed with the control or dabigatran.

### Anticoagulants Alter Unevenly Distributed Fibrin Networks

**Fig. 5:**
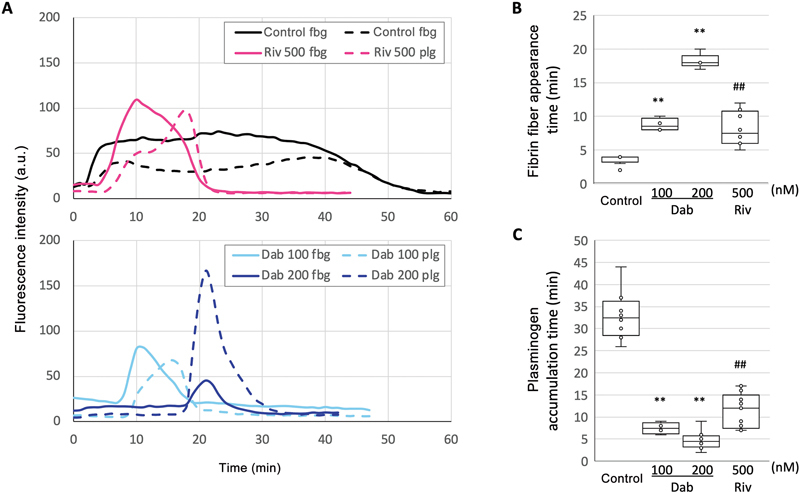
Changes in fluorescence intensity of Alexa Fluor (AF) 488-labeled fibrinogen (fbg-488) and AF 568-labeled plasminogen (plg-568) in the dense fibrin regions. ( **A** ) Fluorescence intensities of both fbg-488 and plg-568 in the central region of five concentric circles placed in the dense fibrin regions were separately measured using ImageJ software. The changes in fluorescence intensity are shown as solid lines (fig-488) and dotted lines (plg-568) without anticoagulants (control, black), or with 500 nM rivaroxaban (Riv 500, pink lines) or 100/200 nM dabigatran (Dab 100/Dab 200, light blue/blue). Fibrin fiber appearance time ( **B** ) and plasminogen accumulation time ( **C** ) were determined by measuring the time, as described in the “Fluorescence Intensity Analysis” section of the Materials and Methods. The fluorescence intensities of both fbg-488 and plg-568 were separately measured in five concentric circles within the dense fibrin network region. Statistical analysis was performed using the Mann–Whitney U test (## *p* < 0.01 vs. control). The dose-dependent effect of the anticoagulants was analyzed using the Shirley–Williams method, a nonparametric multiple comparison test (** *p* < 0.01) from 4 to 8 regions in two to four independent experiments.

**Fig. 6:**
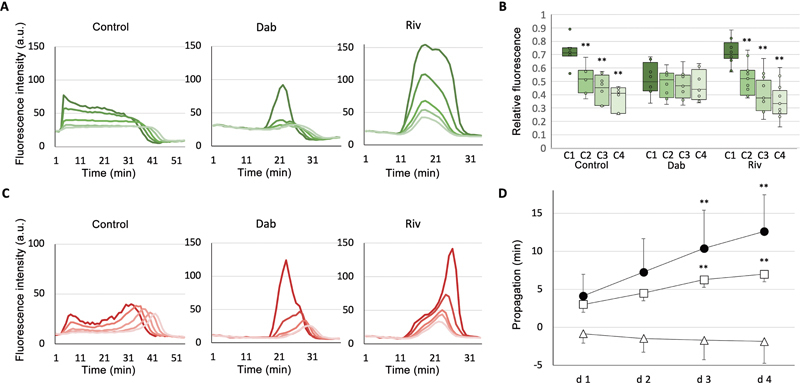
Propagation of fibrin network formation and plasminogen accumulation from the central regions in the dense fibrin to the periphery. The fluorescence intensities of both Alexa Fluor (AF) 488-labeled fibrinogen (fbg-488) and AF 568-labeled plasminogen (plg-568) were separately measured in five concentric circles within the dense fibrin network region. The average intensities were determined at the center and in the four outer ring-shaped areas. The green lines of fbg-488 ( **A** ) and the red lines of plg-568 ( **C** ) are thicker and closer to the center of the concentric circles. Without anticoagulants: control; dabigatran: Dab; rivaroxaban: Riv. ( **B** ) The relative fluorescence intensity of fbg-488 in the outer regions compared with the maximum intensity in the center is shown. A thicker green color indicates concentric circles close to the center (C1), which become lighter green as they move toward the periphery (C2–C4). The decrease in intensity (the intensity relative to C0 from C1 to C4) with increasing distance from the center was analyzed using the Shirley–Williams method, a nonparametric multiple comparison test (8–13 areas from three experiments, ** *p* < 0.01). ( **D** ) The delay in plasminogen accumulation from the center to the outer regions of the concentric circles (D: d1–d4), which were calculated based on the time of peak plg-568 fluorescence, is indicated as closed circles (control), open squares (200 nM dabigatran), and open triangles (500 nM rivaroxaban). Statistical analysis was performed on 8 to 13 areas from three independent experiments using the Shirley–Williams method (** *p* < 0.01).

We precisely examined fluctuations in fluorescence intensities within concentric circles, designated as regions of interest (ROIs) spanning from the central dense fibrin region to its periphery ( Supplementary Fig. S1 , available in the online version). Fig. 5A illustrates changes in fluorescence intensities in the central regions. The fibrin fiber appearance time, defined as the initiation of the fbg-488 increase, was dose-dependently prolonged by dabigatran ( Fig. 5B ). A higher concentration of rivaroxaban (500 nM) resulted in a similar prolongation as a lower concentration of dabigatran (100 nM) ( Fig. 5B ), consistent with clotting time ( Fig. 2E ). Analysis of fbg-488 fluorescence propagation using concentric circles in the control effectively revealed an uneven distribution of the fibrin network, 14 28 as indicated by the significant decline in the relative fluorescence of fbg-488 toward the periphery ( Fig. 6A, B ). The presence of dabigatran mitigated this previously observed uneven distribution, resulting in a more uniform pattern ( Fig. 6A, B , Dab). In contrast, rivaroxaban treatment resulted in a fibrin network distribution ( Fig. 6A, B , Riv) similar to that of the control, exhibiting the same unevenness. These discernible alterations in fibrin network distribution suggest variations in inhibitory mechanisms and localization of the respective anticoagulants. Dabigatran targets thrombin activity within the fluid phase, whereas rivaroxaban impedes thrombin generation at activated platelet surfaces.

### Unique Patterns of Plasminogen Accumulation and Fibrinolysis Near Activated Platelets

Plasminogen accumulation, indicated by increased fluorescence intensity of plg-568, slightly intensified in control approximately 5 minutes after the formation of a dense fibrin network on and around activated platelets ( Fig. 5A : black dotted line; Supplementary Fig. S1A [available in the online version]). Plg-568 fluorescence remained constant for approximately 30 minutes before gradually peaking and declining along with fbg-488 fluorescence due to clot lysis. We refer to this sustaining process of plg-568 as “plasminogen accumulation time” ( Supplementary Fig. S1A , available in the online version), indicative of TAFIa activity. Our previous findings showed that TAFIa inhibition completely halted this process, promoting plg-568 accumulation and resulting in prompt clot lysis. 14

Rivaroxaban gradually increased plg-568 fluorescence within the central region of the concentric circles after a short-sustained period ( Fig. 5A , pink dotted line). In contrast, dabigatran induced a rather prompt augmentation without a sustained period, even at a low concentration of 100 nM ( Fig. 5A , light blue dotted line). The plasminogen accumulation time appeared shorter with dabigatran compared with rivaroxaban ( Fig. 5C ), suggesting inadequate TAFI activation in the central region.

With dabigatran at a concentration of 200 nM, plasminogen accumulation sharply increased after fibrin formation in the central region ( Fig. 5A , blue dotted line) and then spread outward, similar to the control ( Fig. 6C , Dab and control; Fig. 6D ). In contrast, rivaroxaban showed a gradual accumulation of plasminogen in the central region ( Fig. 5A , pink dotted line). Surprisingly, faster plasminogen accumulation and dissolution of fibrin clots were observed in the peripheral region compared to the center of the ROI ( Fig. 6C , Riv), as shown by the negative delay in propagation ( Fig. 6D ). TAFIa inhibitor, with or without rivaroxaban, immediately increased plasminogen accumulation and initiated clot lysis in the central region ( Supplementary Fig. S3A , available in the online version), and then propagated plasminogen accumulation to the periphery ( Supplementary Fig. S3B , available in the online version). These findings suggest that TAFI activation is limited to the central region of dense fibrin with dabigatran, whereas it predominantly occurs in the peripheral region with rivaroxaban.

## Discussion

Through real-time imaging analysis of platelet-initiated plasma clot formation and subsequent lysis, we have identified distinct anticoagulation patterns associated with DTIs and FXaIs, such as dabigatran and rivaroxaban, respectively. These patterns significantly influence TAFI activation and fibrinolysis processes.

**Fig. 7:**
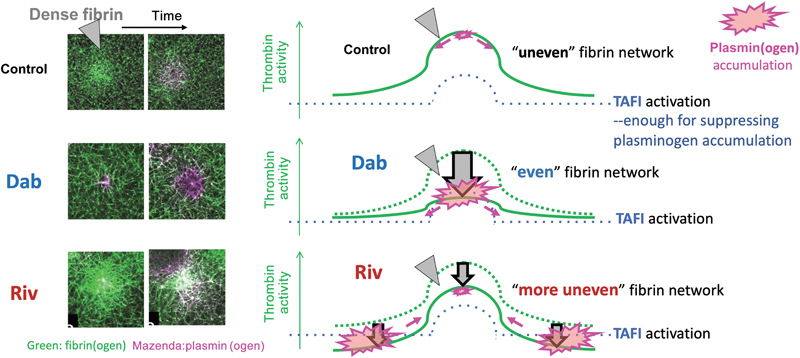
Spatial modulation of thrombin activity influences thrombin-activatable fibrinolysis inhibitor (TAFI) activation and plasminogen accumulation dynamics. The activation of the coagulation cascade, initiated at the surface of activated platelets, results in the formation of an uneven fibrin network with a heterogeneous distribution of thrombin activity (shown by the green curve in control). Dabigatran (Dab), functioning as a direct thrombin inhibitor within the fluid, reduces thrombin activity, particularly in the protruding part of the green line, leading to uniform thrombin activity and a more homogenous fibrin network. In contrast, rivaroxaban (Riv) selectively impedes activated factor X-dependent catalysis of thrombin generation on activated platelet surfaces, resulting in a green curve (representing thrombin activity) with a structure similar to that of the control but with reduced amplitude. This leads to the formation of a more heterogeneous fibrin network. Furthermore, the spatial modification of thrombin activity significantly affects TAFI activation, as indicated by the reduced accumulation of plasminogen. Dotted blue lines represent the threshold of TAFI activation necessary to effectively inhibit plasminogen accumulation when the thrombin activity surpasses these lines. In cases where the thrombin activity (solid green line) falls below the requisite level for TAFI activation (dotted blue line), plasminogen accumulates rapidly, as indicated by the magenta area. This rapid accumulation of plasminogen is commonly observed in the central region of the dense fibrin with Dab and at the outer edges with Riv. The magenta arrows show the direction in which the plasminogen accumulation is spreading.

Our previous investigations have consistently demonstrated the crucial role of activated platelet surfaces, by exposing PS on their outer membrane surface, in initiating the coagulation cascade and promoting fibrin network formation. This phenomenon has been consistently observed both *in vivo* 34 and *in vitro* . 27 PS, an anionic phospholipid, facilitates the aggregation of vitamin K-dependent coagulation factors such as FX and prothrombin by binding to their Gla domains, thereby facilitating their subsequent activation. Rivaroxaban selectively impeded FXa-dependent catalysis of thrombin generation on activated platelet surfaces, leading to irregular distribution of thrombin activity and fibrin network architectures. In contrast, dabigatran reversibly inhibits thrombin activity in the fluid phase, leaving a certain concentration of free thrombin, which results in a uniformly distributed thrombin activity. Once the level of generated thrombin surpasses a certain threshold, a uniform fibrin network structure is formed. Fig. 7 schematically depicts this concept, supported by our real-time imaging analysis.

The uneven distribution of thrombin activity and fibrin networks, along with their alteration by various DOACs, significantly impacts TAFI activation. In Fig. 7 , the green curves represent thrombin activity estimated from the fluorescence intensity of fibrin fibers. Rivaroxaban selectively inhibits FXa on the surfaces of the activated platelets (dense fibrin region), suppressing thrombin generation and maintaining a distribution pattern of thrombin activity (Riv: solid green curve) comparable to that of the control (Riv: dotted green curve). In contrast, dabigatran inhibits thrombin in a fluid phase, generating a uniformly dispersed constant thrombin activity (Dab: solid green curve). Increased plasminogen accumulation following DOAC treatment suggests insufficient TAFI activation in the central (dabigatran) and peripheral (rivaroxaban) regions with rivaroxaban. This implies that the dotted blue line in Fig. 7 illustrates the threshold of TAFI activation required to sufficiently impede plasminogen accumulation when thrombin activity surpasses these lines. The central area also has the highest concentration of substrates for both plasmin and TAFIa. When thrombin activity (solid green line) falls below the requisite level for TAFI activation (dotted blue line), plasminogen accumulates rapidly, as indicated by the red area, particularly in the central region of dense fibrin with dabigatran and at the outer edges with rivaroxaban. This concept introduces, for the first time, a discernible contrast between DTIs and FXaIs regarding plasminogen accumulation and fibrinolysis modulation. Activated platelet-secreted molecules and platelet-dependent mechanical force, 35 along with TAFI activation, influence the dynamics of fibrin network formation and lysis. Consideration should also be given to how DTIs inhibit thrombin activity, affecting their ability to bind thrombomodulin and activate platelets, thereby influencing both coagulation and fibrinolysis.

Numerous studies have examined the impact of anticoagulants on the structure of fibrin networks. 24 36 37 38 Three-dimensional confocal microscopic images of fibrin networks in platelet-poor plasma have shown that anticoagulants result in fewer branched fibers and larger pores, leading to increased permeability of the fibrin gel. However, both DTIs and FXaIs form more porous fibrin clots compared with the control. 37 Similarly, FXaI-treated pooled plasma also produces less dense and more permeable clots. 38 Considering thrombotic disease-related modifications in fibrin clot structure, such as decreased clot permeability in coronary artery disease 39 and idiopathic venous thromboembolism, 40 as well as elevated prothrombin-dependent thin and densely packed fibrin clots, 41 further investigation is required under pathological conditions to understand the differences in the distribution of procoagulant platelet-altered fibrin networks induced by anticoagulants.

Extensive evaluations have compared anticoagulants, primarily using thrombin generation assays. The direct continuous measurement technique of fluorogenic substrate-dependent thrombin activity, referred to as a “global coagulation assay,” provides more comprehensive information on hemostatic potential than conventional clotting assays. 42 Shaw et al clearly illustrated the distinctive patterns of concentration-dependent changes in the thrombin generation curve between direct FXaIs and DTIs, utilizing information from previous reports. 33 This review suggests that FXaIs and DTIs have distinct effects on coagulation initiation and propagation. FXaIs tend to produce a flat, prolonged curve with a suppressed peak but preserved ETP, whereas DTIs delay the onset of thrombin generation with a sharp, right-shifted curve. In our study, using platelet-containing plasma, we confirmed these distinct patterns for both rivaroxaban and dabigatran, although rivaroxaban had a relatively limited effect on clotting time. These differences in thrombin generation curves, coupled with disparities in functioning regions noted in our imaging analysis, contribute to our understanding of the efficacy and safety of these drugs.

Thrombomodulin plays a unique role in regulating both coagulation and fibrinolysis. 43 44 45 This multifunctional molecule can modulate thrombin activity, switching it from a procoagulant state to an anticoagulant and antifibrinolytic state by directly binding to thrombin and activating protein C and TAFI. The effectiveness of this regulatory mechanism depends on the concentrations and spatial distributions of thrombomodulin and thrombin activity. 46 47 48 In our study, we found that soluble thrombomodulin in the plasma effectively activates TAFI, which was dependent on the potentiation of thrombin generation by activated platelets. We observed a positive correlation between lysis time and thrombin generation, which disappeared upon the addition of TAFIaI. This suggests that enhancement of thrombin generation by activated platelets may efficiently activate TAFI. However, a neutralizing antibody against thrombomodulin, aimed at disrupting thrombin binding, did not influence thrombin generation, indicating that protein C activation was not significantly involved, as previously demonstrated. 14 Therefore, we investigated the effects of anticoagulants on fibrinolysis under these experimental conditions without adding thrombomodulin externally.

Based on our spatiotemporal observations, the impact of anticoagulants on fibrinolysis mainly relies on TAFI activation through thrombin activity distribution. Different classes of DOACs modify this activation differently. Lisman et al studied the profibrinolytic potential of anticoagulants, particularly those targeting FXa. They found that targeting FXa had a stronger effect on thrombin generation after fibrin clot formation compared to thrombin or TF in an *in vitro* assay. 20 Conversely, Semeraro et al demonstrated that only DTIs, but not FXaIs, reduced resistance to fibrinolysis due to insufficient TAFI activation. They analyzed plasma samples from patients with atrial fibrillation before and after anticoagulant administration. 22 Other studies 23 24 have also shown the need for thrombin activity to activate TAFI, but only in the presence of recombinant thrombomodulin in the lysis assay. Considering the previous research, we proposed the following points: (1) Soluble thrombomodulin is effective in activating TAFI in the presence of anticoagulants during the lysis of platelet-containing plasma clots. (2) The uneven and patchy structure of the fibrin network formed under normal conditions might be crucial for the hemostatic thrombus properties. (3) Rivaroxaban, an upstream inhibitor of thrombin generation, appeared to help maintain an uneven distribution of thrombin activity and partial activation of TAFI necessary for hemostatic thrombus stability. (4) Dabigatran directly inhibits thrombin activity and strongly prolongs fibrin formation but maintains thrombin generation. Disappearance in the heterogeneous distribution of thrombin activity seems to disrupt the local activation of TAFI, leading to the fragile hemostatic thrombus. Maintaining physiological thrombus configuration having dense fibrin and less fibrinolytic activity on activated platelets even under anticoagulation by rivaroxaban may be advantageous to protect hemostatic thrombi from immature lysis and to avoid the associated bleeding in a clinical setting.

In conclusion, real-time imaging of the formation of an activated platelet-initiated fibrin network, plasminogen accumulation, and fibrinolysis, coupled with spatiotemporal analysis of their patterns, provides novel insights into the specific effects of various classes of anticoagulants on coagulation and fibrinolysis. The distinct impacts of DTIs and FXaIs on TAFI activation, in terms of spatial and temporal regulation, may shed light on variations in bleeding risk associated with anticoagulant therapy using different DOACs. Further studies under flow conditions and through intra-vessel analysis are warranted to confirm this.

## Notes

**What is known about this topic?**

- The effect of direct oral anticoagulants (DOACs) on thrombin generation assays reveals a distinct pattern of changes in coagulation activity.
- Decreased thrombin activity by anticoagulant treatment affects the activation of thrombin-activatable fibrinolysis inhibitor (TAFI), resulting in the facilitation of fibrinolysis.

**What does this paper add?**

- Inhibition of activated factor X or thrombin showed distinct fibrin network formation, impaired activation of TAFI, and spatially different subsequent lysis patterns, which were identified through confocal microscopy analysis using human platelet-containing plasma.
- Visualization analysis revealed the spatiotemporal effects of DOACs on fibrinolytic activity. This could explain the variations in how different anticoagulants impact the stability of hemostatic thrombi, potentially affecting the risk of bleeding complications.
