# Supplementary material for: Real-Time Imaging of Platelet-Initiated Plasma Clot Formation and Lysis Unveils Distinct Impacts of Anticoagulants
Source: Thromb Haemost. 2025 Jan 9;125(8):766–78. doi: 10.1055/a-2497-4213 (PMC12283144; doi:10.1055/a-2497-4213)
Supplement: Supplementary file 1 — Supplementary Material [file 10-1055-a-2497-4213-s24090461.pdf]

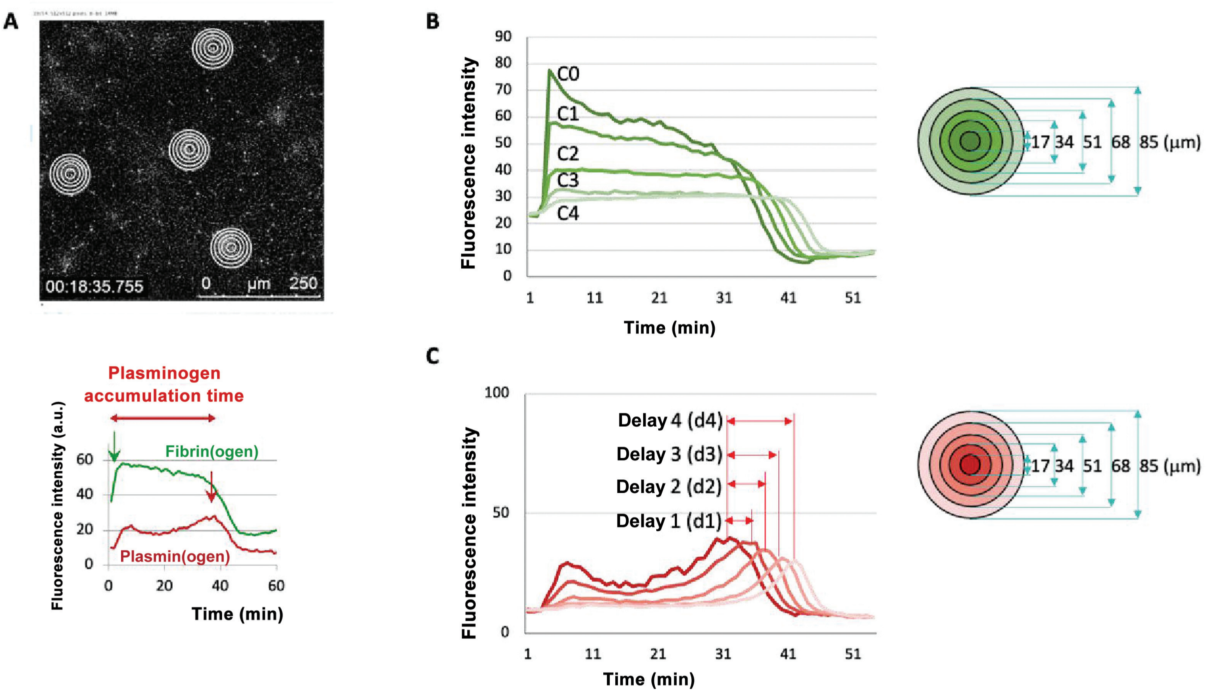

Supplementary Fig. S1 Fluorescence intensity analysis of fibrin network and plasminogen accumulation around activated platelets. (**A**) Five concentric circles were manually drawn in the region centered on the dense fibrin network based on the fluorescence of Alexa Fluor (AF) 488-labeled fibrinogen. Captured images were obtained from the same video, as shown in ►**Fig. 4A**. The concentric circles have diameters of 15, 30, 45, 60, and 75 pixels (approximately 17, 34, 51, 68, and 85 μm, respectively). Changes in the fluorescence intensity of AF 488-labeled fibrinogen (green) and AF 568-labeled plasminogen (red) in the central region of the concentric circles were traced and graphed. The green arrow indicates the beginning of the increase in green fluorescence, defined as the fibrin fiber appearance time. The red arrow indicates an increase in the maximum level of red fluorescence. The time from the green arrow to the red arrow was defined as the plasminogen accumulation time. (**B**) Representative changes in the green fluorescence intensity in each concentric region from the center (C0) to the periphery (C4) are shown. (**C**) Representative changes in the red fluorescence intensity and propagation from the center to the periphery of the maximum fluorescence intensity in five different regions are shown as delays of 1 to 4.

 © 2025. The Author(s).

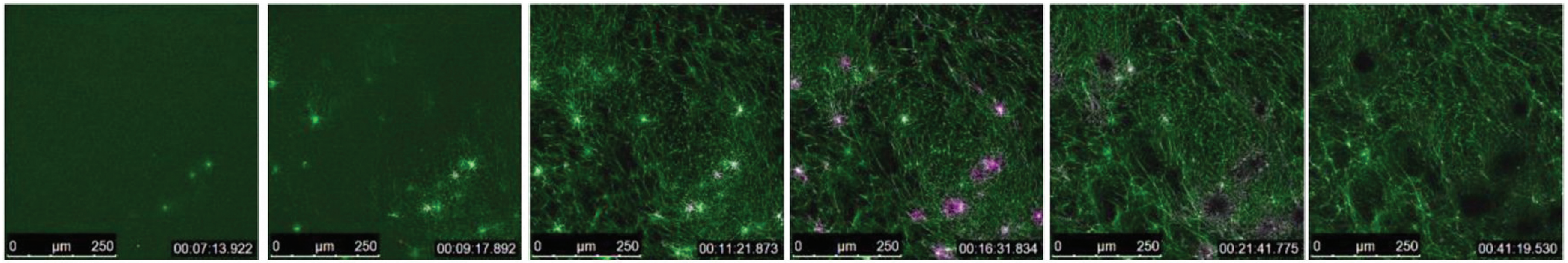

Supplementary Fig. S2 Clot formation and dissolution in platelet-containing plasma in a low concentration of dabigatran. The sequential overlaid images obtained via confocal laser scanning microscopy (TCS SP8; Leica Microsystems GmbH) are displayed. Coagulation and fibrinolysis of platelet-containing plasma, supplemented with a low concentration of 100 nM dabigatran, were initiated through tissue factor and tissue-type plasminogen activator, respectively. Trace amounts of Alexa Fluor (AF) 488-labeled fibrinogen (green) and AF 568-labeled plasminogen (magenta) were added for visualization. Images captured 7, 9, 11, 16, 21, and 40 minutes after initiation are shown.

 © 2025. The Author(s).

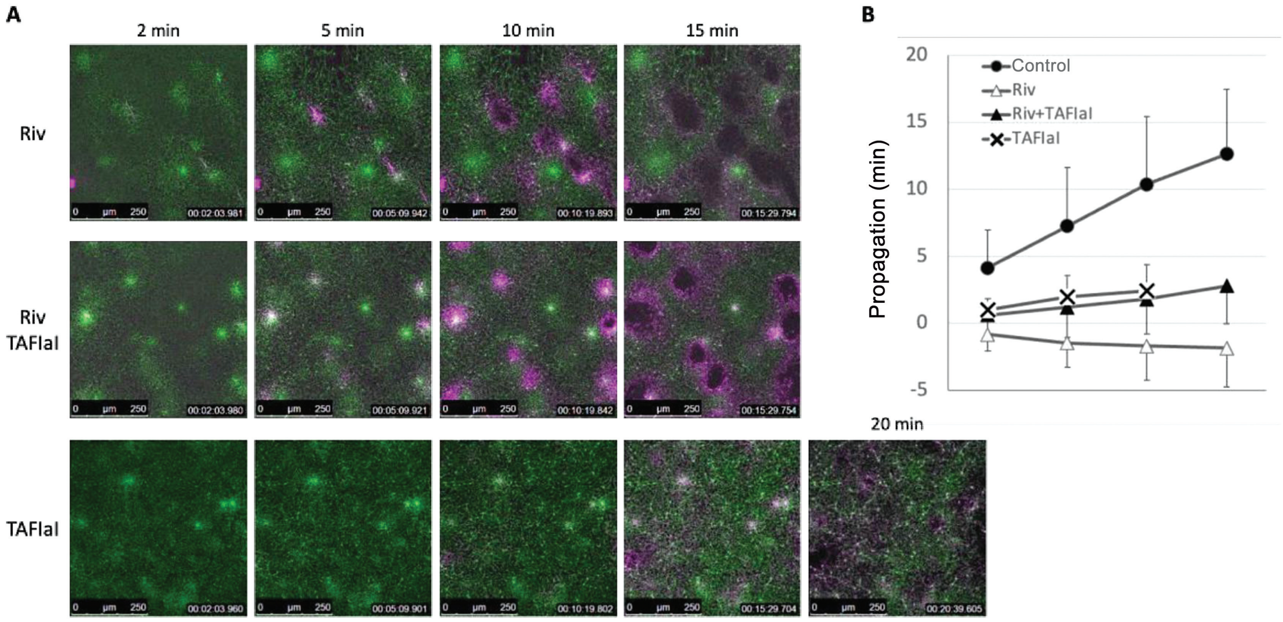

Supplementary Fig. S3 Rivaroxaban activates thrombin-activatable fibrinolysis inhibitor (TAFI) in the dense fibrin region and demonstrates resistance to fibrinolysis in platelet-containing plasma clot formation and lysis analysis. (**A**) Sequential overlaid images of rivaroxaban 500 nM (Riv) and/or TAFIa inhibitor (TAFIaI) 5 µM are displayed 2, 5, 10, 15, and 20 minutes after the capture. Green: Alexa Fluor (AF) 488-labeled fibrinogen; Magenta: AF 568-labeled plasminogen; bars: 50 µm. (**B**) Delays in plasminogen accumulation peak values (d1 to d4) calculated from five concentric circles from the dense fibrin network to the outer region are shown. Data are presented as the mean plus or minus the standard deviation.

 © 2025. The Author(s).
